# Anti-Influenza Activity of C_60_ Fullerene Derivatives

**DOI:** 10.1371/journal.pone.0066337

**Published:** 2013-06-13

**Authors:** Masaki Shoji, Etsuhisa Takahashi, Dai Hatakeyama, Yuma Iwai, Yuka Morita, Riku Shirayama, Noriko Echigo, Hiroshi Kido, Shigeo Nakamura, Tadahiko Mashino, Takeshi Okutani, Takashi Kuzuhara

**Affiliations:** 1 Laboratory of Biochemistry, Faculty of Pharmaceutical Sciences, Tokushima Bunri University, Yamashiro-cho, Tokushima, Japan; 2 Division of Enzyme Chemistry, Institute for Enzyme Research, The University of Tokushima, Tokushima, Japan; 3 Department of Chemistry, Nippon Medical School, Nakahara-ku, Kawasaki, Kanagawa, Japan; 4 Department of Pharmaceutical Sciences, Faculty of Pharmacy, Keio University, Minato-ku, Tokyo, Japan; University of Edinburgh, United Kingdom

## Abstract

The H1N1 influenza A virus, which originated in swine, caused a global pandemic in 2009, and the highly pathogenic H5N1 avian influenza virus has also caused epidemics in Southeast Asia in recent years. Thus, the threat from influenza A remains a serious global health issue, and novel drugs that target these viruses are highly desirable. Influenza A RNA polymerase consists of the PA, PB1, and PB2 subunits, and the N-terminal domain of the PA subunit demonstrates endonuclease activity. Fullerene (C_60_) is a unique carbon molecule that forms a sphere. To identify potential new anti-influenza compounds, we screened 12 fullerene derivatives using an *in vitro* PA endonuclease inhibition assay. We identified 8 fullerene derivatives that inhibited the endonuclease activity of the PA N-terminal domain or full-length PA protein *in vitro*. We also performed *in silico* docking simulation analysis of the C_60_ fullerene and PA endonuclease, which suggested that fullerenes can bind to the active pocket of PA endonuclease. In a cell culture system, we found that several fullerene derivatives inhibit influenza A viral infection and the expression of influenza A nucleoprotein and nonstructural protein 1. These results indicate that fullerene derivatives are possible candidates for the development of novel anti-influenza drugs.

## Introduction

In 1918, an influenza A pandemic caused 50 million deaths worldwide [1], and the development of strategies that can be used to prevent future expansions of this virus continues to be an important endeavor [2]. The avian H5N1 influenza A virus is highly pathogenic to humans [3], and the emergence of a new strain of this virus in 2009, the swine-originating A/H1N1 pdm influenza virus, further emphasizes that this issue is a serious global health problem [4], [5]. Although inhibitors of influenza A, e.g., the neuraminidase-like compound oseltamivir, are widely used as antiviral drugs [6], [7], the adverse effects of these agents and the emergence of viral strains that are resistant to these drugs have now been reported [8], [9].

To prevent and control influenza outbreaks, the development of novel antiviral drugs that are not based on neuraminidase inhibition is now regarded as critical. The influenza A genome consists of segmented single-stranded RNA (-), and its transcription and replication require the activity of a highly conserved RNA-dependent RNA polymerase [10], [11]. This polymerase is essential for the propagation of the influenza A virus and is a very promising target for the development of antiviral drugs. The influenza A virus RNA polymerase is composed of three subunits–PA, PB1, and PB2 [12]–and synthesizes viral mRNA using short capped primers that are cleaved from the host’s cellular pre-mRNAs by the viral endonuclease [13], [14]. Yuan et al. and Dias et al. have shown that the N-terminal domain of the PA subunit contains the active site of the endonuclease, and that this domain also harbors RNA/DNA endonuclease activity [13], [14]. Hence, we speculate that PA endonuclease would contain very effective targets for the development of novel anti-influenza A drugs, as we have shown that several chemicals, e.g., catechins, phenethylphenyl phthalimide analogs, and marchantin analogs, inhibit this endonuclease and possess antiviral activity [15]–[17].

**Figure 1:**
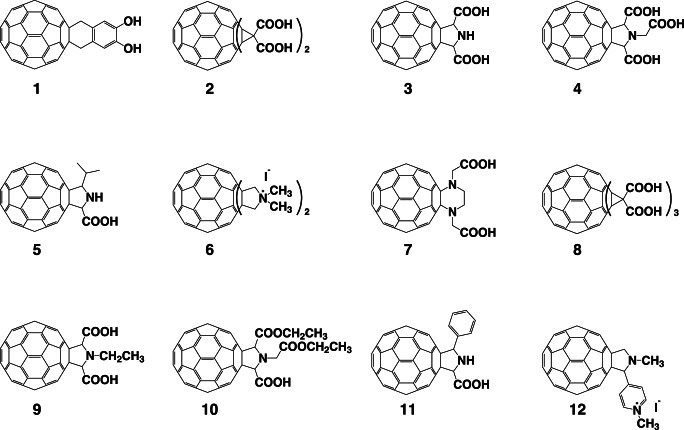
Chemical structures of the C60 fullerene derivatives tested in this study. The chemical structures of the fullerene derivatives examined in this study are shown. The sources for these structures are described in the Materials and Methods. No. 1, 1,4-dihydro-6,7-dihydroxy [60]fullerenonaphthlene; no. 2, [60]fullerenodicyclopropane-1,1,1′,1′-tetracarboxylic acid; no. 3: [60]fullerenopyrrolidine-2,5-dicarboxylic acid; no. 4, 1-carboxymethyl [60]fullerenopyrrolidine-2,5-dicarboxylic acid; no. 5, 5-isopropyl [60]fullerenopyrrolidine-2-carboxylic acid; no. 6∶1,1,1′,1′-tetramethyl [60]fullerenodipyrrolidinium diiodide; no. 7, [60]fullerenopiperazine-1,4-diacetic acid; no. 8: [60]fullerenotricyclopropane-1,1,1′,1′,1′′,1′′-hexacarboxylic acid; no. 9, 1-ethyl [60]fullerenopyrrolidine-2,5-dicarboxylic acid; no. 10, 1-ethoxycarbonylmethyl [60]fullerenopyrrolidine-2,5-dicarboxylic acid 2-ethyl ester; no. 11, 5-phenyl [60]fullerenopyrrolidine-2-carboxylic acid; and no. 12, 4-(1′-methyl [60]fullerenopyrrolidin-2′-yl)-1-methylpyridinium iodide.

Fullerene (C_60_), a carbon buckyball, was discovered by Harold Kroto, James R. Heath, Sean O’Brien, Robert Curl, and Richard Smalley in 1985 [18]. It has since been utilized in electronic and mechanical applications [19]. In physiological studies, the biological effects of water-soluble fullerene derivatives containing several hydrophilic groups are noteworthy because fullerene itself is water-insoluble. Water-soluble fullerene derivatives are known to possess various biological and pharmacological properties, including antioxidant activity and inhibitory effects against human immunodeficiency virus (HIV) proteases and DNA photocleavage [20]–[23]. Mashino et al. also demonstrated that pyrrolidinium fullerene derivative 6 (Fig. 1) has antiproliferative and antibacterial activity [24], malonic acid fullerene derivative 2 (Fig. 1) has excellent antioxidant activity [25], and proline-modified fullerene derivative 3 (Fig. 1) inhibits HIV-reverse transcriptase [22]. Thus, fullerene derivatives are expected to become a novel type of medication because of their unique skeleton.

In our current study, we used an *in vitro* influenza PA endonuclease assay to analyze the effects of 12 different fullerene derivatives on the endonuclease activity of the PA N-terminal domain and full-length PA. We found that the fullerene derivatives inhibit influenza PA endonuclease activity and viral infection. Our results indicate the possibility of developing fullerene derivatives into novel anti-influenza A drugs in the future.

## Results

### Inhibition of PA Endonuclease by Fullerene Derivatives

**Figure 2:**
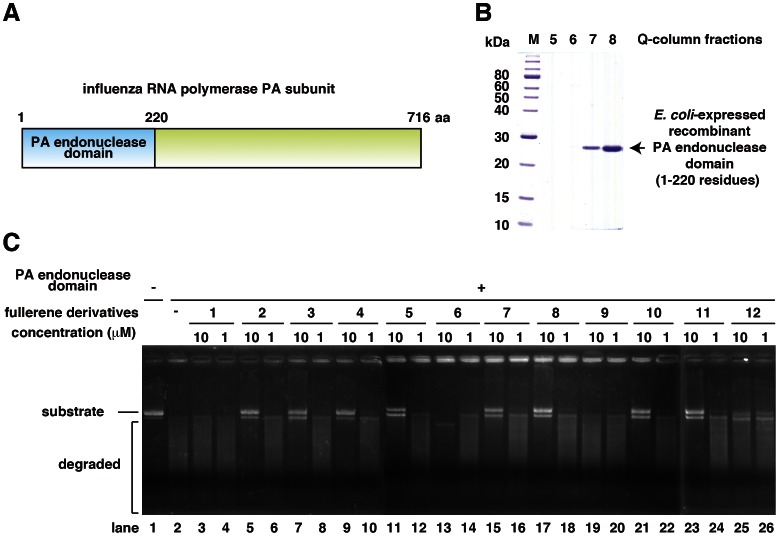
Inhibition of the activity of the PA endonuclease domain by the fullerene derivatives. (**A**) Schematic of the PA subunit of influenza RNA polymerase. (**B**) Purification of the bacterially expressed PA endonuclease domain using a HiTrap-Q column. The arrow indicates the PA endonuclease domain. (**C**) The effects of the various fullerene derivatives on the endonuclease activity of the PA N-terminal domain of the influenza A RNA polymerase were tested. The recombinant PA N-terminal domain protein was added to each reaction at a concentration of 0.25 µg/100 µL. A zero control (i.e., no PA domain added) was also assayed. The fullerene derivatives were added at a dose of 1 or 10 µM, and M13 mp18 was used as the substrate.

For the *in vitro* PA endonuclease assay, we expressed and purified a recombinant influenza PA endonuclease domain (1–220 residues; Fig. 2A, B) using bacteria as described previously [15]–[17]. For the assay, we incubated 0.1 µM recombinant PA endonuclease domain with 1 or 10 µM of each fullerene derivative (Fig. 2C). The PA endonuclease domain digested M13 mp18 circular single-stranded DNA *in vitro* (Fig. 2C, lanes 1 and 2) [12]–[16], and we investigated whether any of the fullerene derivatives could inhibit this activity. The fullerene derivatives 2–5, 7, 8, 10, and 11 significantly inhibited the digestion of M13 mp18 at a dose of 10 µM, no. 12 slightly inhibited digestion, and no. 1, 6, and 9 had no or weak inhibitory activity (Fig. 2C). This is the first study to report that fullerene derivatives can inhibit the activity of influenza enzymes. Fullerene derivative no. 6 caused a mobility shift of M13 mp18, possibly because it has a cationic amine group (Fig. 2C, lane 13). The solubility of the fullerene derivatives 1 and 6 in water was relatively low, which might have been the cause of their decreased activity levels.

**Figure 3:**
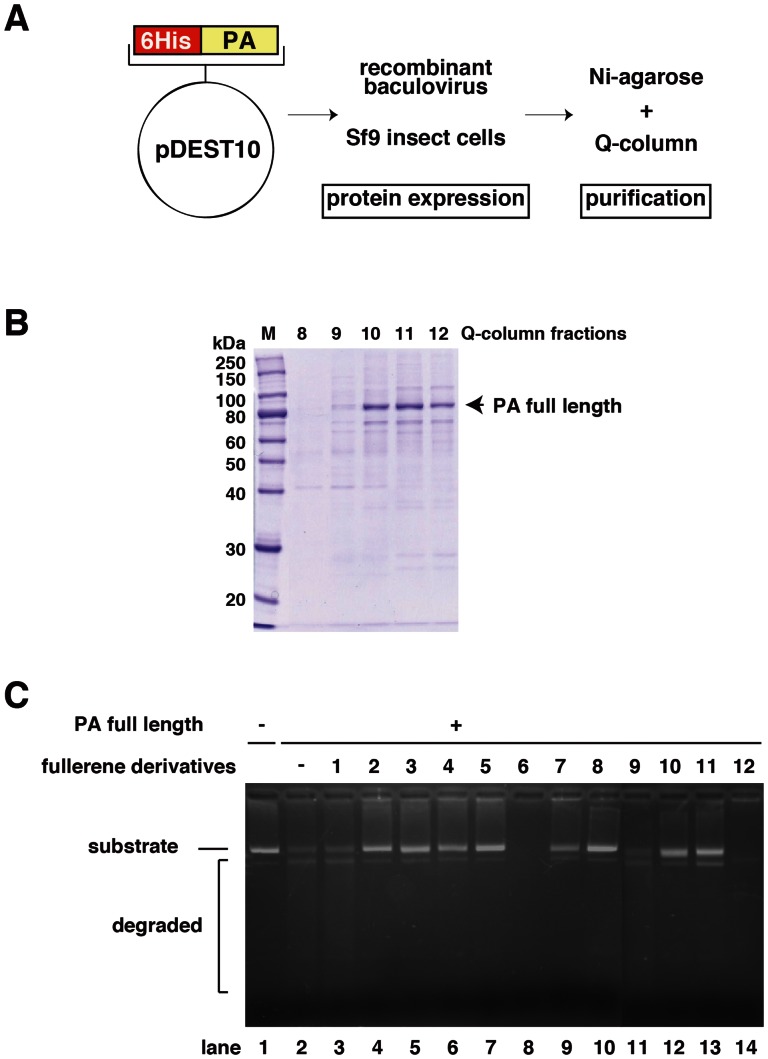
Inhibition of the activity of full-length PA endonuclease by the fullerene derivatives. (**A**) Schematic of the constructed plasmid, baculovirus expression, and purification of full-length PA protein. (**B**) Purification of full-length PA protein using a HiTrap-Q column. The numbers indicate the fractions. The arrow indicates full-length PA protein. (**C**) The effects of the various fullerene derivatives on the endonuclease activity of full-length PA protein of influenza A RNA polymerase were tested. Recombinant full-length PA protein was added to each reaction at a concentration of 0.25 µg/100 µL. A zero control (i.e., no PA protein added) was also assayed. The fullerene derivatives were added at a dose of 10 µM and M13 mp18 was used as the substrate.

To investigate the effects of full-length PA protein on PA endonuclease activity and the inhibitory activity of the fullerene derivatives, we examined whether the fullerene derivatives also inhibit the endonuclease activity of full-length PA protein. We expressed the recombinant full-length PA protein by using a baculovirus to infect Sf9 insect cells (Fig. 3A) and purified it using a Ni-agarose and HiTrap-Q column (Fig. 3B). The recombinant full-length PA protein demonstrated endonuclease activity (Fig. 3C, lanes 1 and 2) [26], [27]. Then, we tested the 12 fullerene derivatives using this same assay. The fullerene derivatives 2–5, 7, 8, 10, and 11 inhibited the endonuclease activity of full-length PA (Fig. 3C), which is consistent with the results for the PA endonuclease domain (Fig. 2C). The M13 mp18 band in the no. 12-treated lane was degraded (Fig. 3C lane 14), the band in the no. 1 and 9-treated lanes slightly remained (Fig. 3C, lanes 3 and 11), suggesting that fullerene derivatives no. 1 and 9 also slightly inhibited the PA endonuclease activity of the full length PA protein.

**Figure 4:**
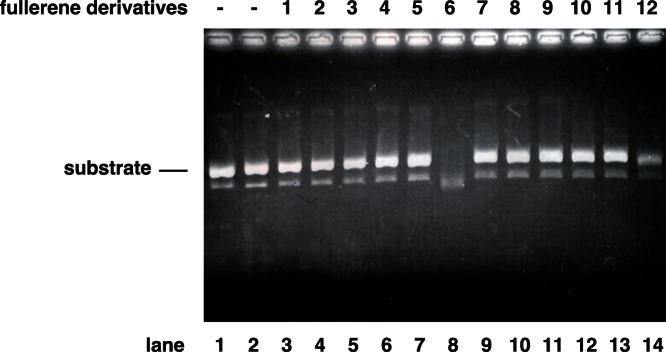
Nuclease activity of the fullerene derivatives. The method was the essentially same as that of Figs. 2 & 3, except the condition of the absence of PA protein. The fullerene derivatives were added at a dose of 10 µM and M13 mp18 was used as the substrate. The digestion of the substrate was examined by agarose electrophoresis.

As shown in Figs. 2C and 3C, the M13 mp18 band in the no. 6-treated lanes shifted and was clear (Fig. 3C lane 8), respectively, suggesting that no. 6 has the ability to cleave DNA. Thus, we examined the nuclease activity of the fullerene derivatives (Fig. 4). The result showed that fullerene derivative no. 6 by itself has significant nuclease activity in the absence of PA endonuclease (Fig. 4). No. 12 also showed weaker nuclease activity by itself (Fig. 4).

### Docking Simulation of Influenza A Endonuclease and C_60_ Fullerene

**Figure 5:**
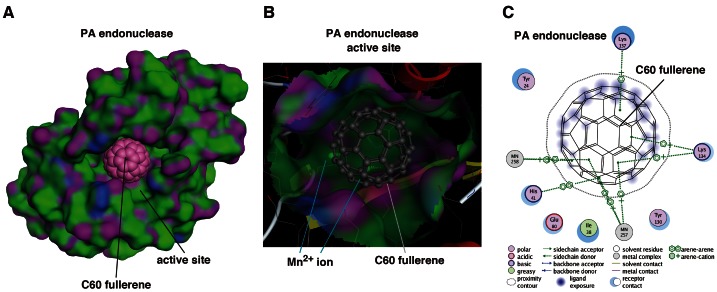
Docking simulation of C_60_ fullerene with influenza PA endonuclease. (**A**) Docking simulation analysis of C_60_ fullerene with the PA endonuclease domain of influenza A RNA polymerase. The fullerene is shown as a sphere. The surface of the pocket of PA endonuclease is shown in green and purple. The pink ball indicates the carbon atoms in the fullerene. (**B**) The fitting of the fullerene to the active pocket of PA endonuclease. PA endonuclease is depicted as a ribbon structure. The α-helix and β-strands are shown in red and yellow, respectively. The fullerene is shown as a gray stick structure. The manganese ions in PA endonuclease are behind the fullerene. (**C**) Two-dimensional analysis of the interactions between fullerene and PA endonuclease. The fullerene is shown in the center with the key and with the interacting amino acids shown around it. MN indicates the Mn^2+^ ions. The modes of interaction are shown at the bottom. The arene of the fullerene interacts with 2 Mn^2+^ ions and the amino acids, e.g., lysine and histidine, in PA endonuclease.

Previously, we reported that 3 of 34 phthalimide chemicals and 5 of 33 phytochemicals inhibited PA endonuclease activity [12], [15], [16]. In the case of the fullerenes, 8 of the 12 fullerene derivatives inhibited PA endonuclease activity; thus, we thought that the fullerene skeleton itself could fit into the active pocket of the influenza PA endonuclease domain. To investigate how the fullerene molecule binds to and fits in the active pocket of the PA endonuclease domain, we performed *in silico* docking simulation analysis of this interaction at the level of the tertiary structure using Molecular Operating Environment software (MOE; Chemical Computing Group, Quebec, Canada) [28]. The results show that fullerene fits into and fills the active pocket of the endonuclease domain of the influenza RNA polymerase (Fig. 5A), suggesting that this may be the major cause of the inhibitory mechanism. The two divalent ions of manganese in the active pocket are reportedly necessary for influenza endonuclease activity [13], [14]. Fullerene binds to manganese ions by arene-cation interactions at the back of the active pocket (Fig. 5B and 5C), suggesting that this binding is also one of the inhibitory mechanisms.

### Toxicity of the Fullerene Derivatives Against the Madin-Darby Canine Kidney Cell Line

**Figure 6:**
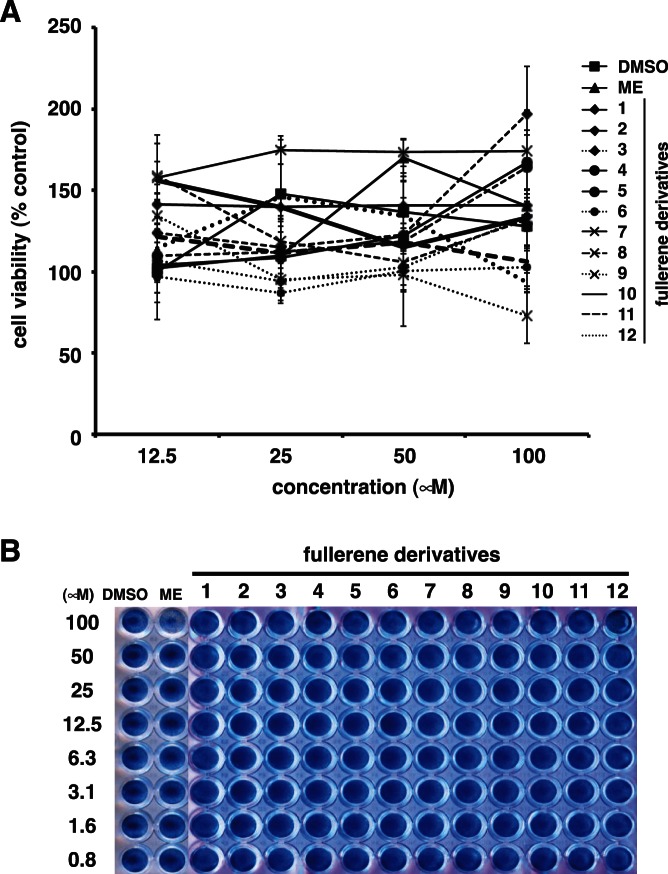
Toxicity of the fullerene derivatives against MDCK cells. (**A**) Various concentrations (12.5–100 µM) of the fullerene derivatives (n = 4) were added to cultures of MDCK cells. DMSO and ME were used as negative and positive controls for anti-influenza activity, respectively. At 24 h post-incubation, cell viability was determined using an MTT cell proliferation assay. Data represent the mean ± standard error of the mean (S.E.M.). (**B**) Various concentrations (0.8–100 µM) of the fullerene derivatives were added to cultures of MDCK cells. ME was used as positive control for cytotoxicity. At 24 h post-incubation, the cells were fixed and viable cells were stained with a naphthol blue black solution.

We evaluated the toxicity of the fullerene derivatives against Madin-Darby canine kidney (MDCK) cells before examining their antiviral activity against the influenza A virus. Various concentrations (12.5–100 µM) of the fullerene derivatives were added to cultures of MDCK cells. Marchantin E (ME) was used as the positive controls for anti-influenza activity [16]. At 24 h post-incubation, the cell viability of the treated-cells was determined using an MTT cell proliferation assay (Fig. 6A). The viability of the cells treated with the fullerene derivatives 1–12 and ME was not significantly different to that of the cells treated with dimethyl sulfoxide (DMSO) at a concentration of 12.5 to 100 µM. We also performed naphthol blue black assay for cytotoxicity of fullerene derivatives (Fig. 6B). At 24 h post-incubation, the viable cells were stained using a blue dye. The wells treated with 0.8–100 µM of the fullerene derivatives 1–12 and DMSO were stained blue (Fig. 6B). Taken together, these data show that the fullerene derivatives (1–12) are not toxic to MDCK cells up to a concentration of 100 µM.

### Inhibition of Influenza A Virus Infection by the Fullerene Derivatives

**Figure 7:**
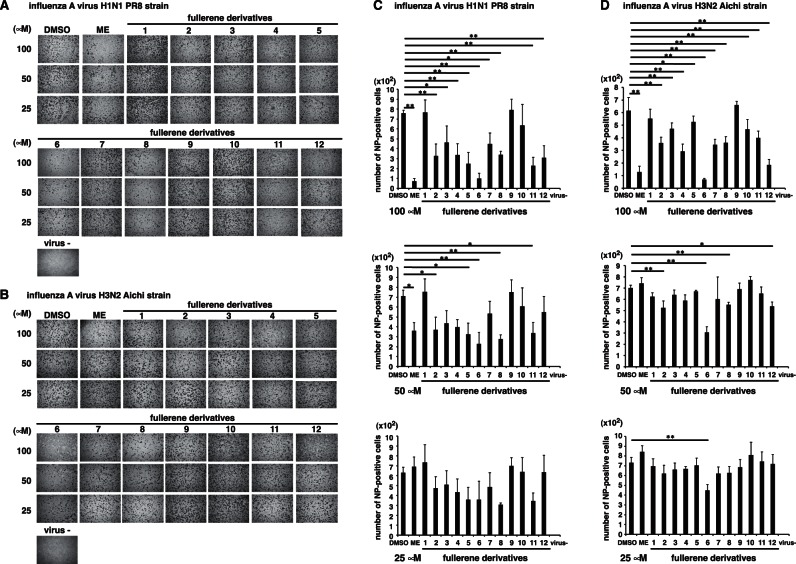
Immunostaining of influenza A virus-infected cells. Various concentrations of the fullerene derivatives (25–100 µM) and an MOI of 1 influenza A virus (A/PR/8/34 (H1N1) (n = 3) (**A and C**) or A/Aichi (H3N2) (n = 4) (**B and D**)) were mixed and added to cultures of MDCK cells. At 24 h post-infection, influenza A NP-immunostaining of the treated cells was performed. The wells were photographed under a microscope (×4) (**A and B**), and the stained cells were counted (**C and D**). DMSO (n = 4) and ME (n = 4) were used as negative and positive controls for the inhibitory effect of influenza A virus infection, respectively. Data represent the mean ± S.E.M. *p<0.05, **p<0.01.

We evaluated the antiviral activity of the fullerene derivatives against the influenza A virus (A/Puerto Rico (PR)/8/34 (H1N1) or A/Aichi/2/68 (H3N2)). Various concentrations of the fullerene derivatives and the virus were mixed and added to cultures of MDCK cells [29]. ME and DMSO were used as positive and negative controls for the inhibitory effect of influenza A virus infection, respectively. At 24 h post-infection, we performed influenza A nucleoprotein (NP)-immunostaining of the treated cells, and the stained cells were counted. At 100 µM, fullerene derivatives no. 2–8, 11 and 12 significantly reduced the number of NP-positive cells in comparison with the control (DMSO), in A/PR8/34 (H1N1)-infected cells (Fig. 7A & C). Also in A/Aichi/2/68 (H3N2)-infected cells, at 100 µM, fullerene derivatives no. 2–8 and 10–12 significantly reduced the number of NP-positive cells in comparison with the DMSO (Fig. 7B & D). The fullerene derivatives 10 in A/PR8/34 (H1N1) also slightly decreased the number of NP-positive cells (Fig. 7A & C). Conversely, the number of NP-positive cells treated with the fullerene derivatives 1 and 9 were comparable to that of the DMSO-treated cells (Fig. 7A–D).

Based on these results, to compare their activities quantitatively, we calculated IC_50_ values of fullerene derivatives against A/PR8/34 (H1N1) and A/Aichi/2/68 (H3N2) strains. Against H1N1 PR8 strain, IC_50_ values are as follows: 57 µM for fullerene derivatives no. 2; 70 µM for no. 4; 37 µM for no. 5; 20 µM for no. 6; 37 µM for no. 8; 44 µM for no. 11; 78 µM for no. 12; more than 100 µM for no. 3, 7 or 10; 43 µM for ME (Table 1). Against H3N2 Aichi strain, IC_50_ values: 91 µM for fullerene derivatives no. 4; 31 µM for no. 6; 63 µM for no. 12; more than 100 µM for no. 2, 3, 5, 7, 8, 10 or 11; 53 µM for ME (Table 1). IC_50_ values of fullerene derivatives no. 1 or 9 could not be calculated against the strains because of their weak activities (Table 1). Taken together, it indicated that several fullerene derivatives have stronger anti-influenza activity than ME.

**Table 1:** IC_50_ of the fullerene derivatives against influenza A virus H1N1 and H3N2 strains.

		IC_50_ (µM)	
		H1N1 PR8 strain	H3N2 Aichi strain
Marchantin E		43	53
Fullerene derivatives	no. 1	ND	ND
	no. 2	57	>100
	no. 3	>100	>100
	no. 4	70	91
	no. 5	37	>100
	no. 6	20	31
	no. 7	>100	>100
	no. 8	37	>100
	no. 9	ND	ND
	no. 10	>100	>100
	no. 11	44	>100
	no. 12	78	63

ND: not detected.

**Figure 8:**
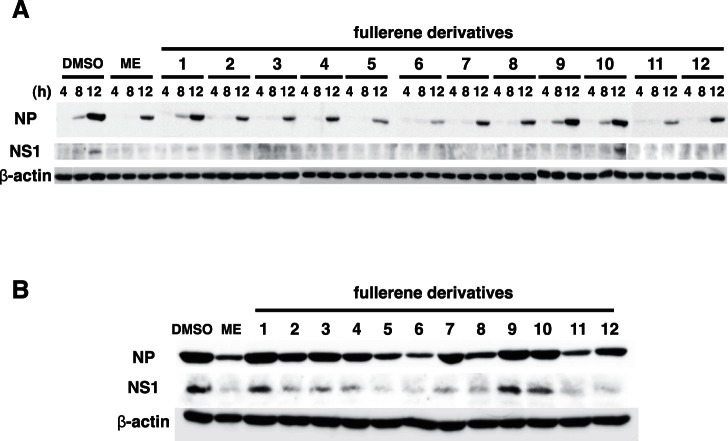
Expression levels of influenza A viral proteins. We mixed 100 µM of the fullerene derivatives or ME and an MOI of 1 influenza A virus (A/PR/8/34 (H1N1)) and added the mixture to cultures of MDCK cells. At 4, 8, 12 (**A**), and 24 h (**B**) postinfection, the expression levels of influenza A NP and NS1 proteins in treated-cell lysates were analyzed by western blotting, and β-actin was analyzed as an internal control. The experiments were performed three times and the results were reproducible.

Moreover, we examined the expression levels of viral proteins by western blotting of treated-cell lysates in A/PR8/34 (H1N1)-infected wells at 4, 8, 12 (Fig. 8A), and 24 h (Fig. 8B) post-infection. The expression levels of influenza A NP and nonstructural protein 1 (NS1) proteins in the cells treated with the fullerene derivatives 5, 6, and 11, and ME were reduced as compared with that of the DMSO-treated cells, but slightly reduced in the wells treated with the fullerene derivatives 2–5, 7, 8, 10 and 12 (Fig. 8A and 8B). Conversely, the expression levels of influenza A NP and NS1 proteins in cells treated with the fullerene derivatives 1 and 9 were comparable to those in the DMSO-treated cells (Fig. 8A and 8B). Taken together, these data show that the fullerene derivatives 2–8 and 10–12 possess antiviral effects against the influenza A virus, and their mechanism of action may be by the inhibition of PA endonuclease activity (no. 2–5, 7, 8, and 11) or their ability to cleave viral RNA (no. 6 and 12).

## Discussion

In this study, we showed that the fullerene derivatives 2–5, 7, 8, 10, and 11 or 6 and 12 possess inhibitory activity against influenza PA endonuclease or the ability to cleave DNA, respectively. Moreover, we showed that the fullerene derivatives 2–8 and 10–12 inhibit the infection of the influenza A virus. Above all, no. 6 showed the strongest antiviral activity. A previous report showed that certain fullerene derivatives have DNA and RNA cleavage activity [20], [30]. As shown in Figs. 2C and 3C, the M13 mp18 band in the no. 6-treated lanes shifted and was clear, respectively. Since no. 6 has the activity to cleave DNA, the antiviral activity of no. 6 may be caused by its cleavage of viral RNA. Therefore, fullerene derivatives are promising novel anti-influenza chemicals. These data are an important advance that could be used in future strategies to refine fullerene-based drug designs. Our analysis provides valuable new information for the design of novel anti-influenza drugs. There was no correlation between the PA endonuclease and antiviral activity of fullerene derivative no. 12. This may be because it targets influenza A virus attachment/entry or growth in cells and also because of differences in its permeability into the cells. When we performed the anti-viral experiment without pre-incubation, we could not find an experimental condition under which the fullerene derivatives showed anti-virus activity. This suggests that the fullerene derivatives may have virucidal activity or they may enter cells by associating with the virus.

Other groups also have reported novel anti-influenza polymerase inhibitors such as T-705 and L-742,001 [27], [31]–[36], which are substituted pyrazine and piperidine compounds, respectively. Since the chemical structures of fullerene derivatives are completely different from those of them, indicating that fullerene derivatives are quite novel anti-influenza compounds.

Finally, we conclude that the chemical and biochemical information presented here will be very useful for the future development of novel fullerene-based drugs against influenza A.

## Materials and Methods

### Preparation of the C_60_ Fullerene Derivatives

Water-soluble fullerene derivatives were synthesized and purified using previously reported methods with small modifications [20]–[25]. All of the fullerene derivatives were dissolved in DMSO to a concentration of 10 mM as stock solutions. The fullerene derivatives (Fig. 1) used in these experiments consisted of the following [20]–[25]: no. 1, 1,4-dihydro-6,7-dihydroxy [60]fullerenonaphthlene; no. 2, [60]fullerenodicyclopropane-1,1,1′,1′-tetracarboxylic acid; no. 3, [60]fullerenopyrrolidine-2,5-dicarboxylic acid; no. 4, 1-carboxymethyl [60]fullerenopyrrolidine-2,5-dicarboxylic acid; no. 5, 5-isopropyl [60]fullerenopyrrolidine-2-carboxylic acid; no. 6, 1,1,1′,1′-tetramethyl [60]fullerenodipyrrolidinium diiodide; no. 7, [60]fullerenopiperazine-1,4-diacetic acid; no. 8, [60]fullerenotricyclopropane-1,1,1′,1′,1′′,1′′-hexacarboxylic acid; no. 9, 1-ethyl [60]fullerenopyrrolidine-2,5-dicarboxylic acid; no. 10, 1-ethoxycarbonylmethyl [60]fullerenopyrrolidine-2,5-dicarboxylic acid 2-ethyl ester; no. 11, 5-phenyl [60]fullerenopyrrolidine-2-carboxylic acid; and no. 12, 4-(1′-methyl [60]fullerenopyrrolidin-2′-yl)-1-methylpyridinium iodide.

### Bacterial Expression and Purification of the PA Endonuclease Domain

The influenza A virus (A/PR/8/34 (H1N1)) RNA polymerase PA plasmid, pBMSA-PA, was obtained from the DNA bank at Riken BioResource Center (Tsukuba, Japan; originally deposited by Susumu Nakada) [37]. The cDNA fragment corresponding to the PA N-terminal endonuclease domain (residues 1–220; Fig. 2A) was amplified by polymerase chain reaction (PCR) [38] from pBMSA-PA. The amplified product was subcloned into the pET28a (+) plasmid (Novagen, Madison, WI, USA). The induction of recombinant protein expression was achieved by the addition of isopropyl-D-thiogalactopyranoside [39], and this was followed by purification using Ni^2+^-agarose [40]. The recombinant PA endonuclease domain protein was further purified to near homogeneity (Fig. 2B) using a HiTrapQ-FF column (GE Healthcare, Buckinghamshire, UK).

### Baculoviral Expression and Purification of the Full-length PA Subunit

The cDNA fragment corresponding to the full-length protein-coding region of PA was amplified by PCR [38] from pBMSA-PA using the primers PA_start_TOPO (CAC CAT GGA AGA TTT TGT GCG AC), PA stop (CTA ACT CAA TGC ATG TGT AAG), PA_mid_anti (TCT TTG GAC ATT TGA GAC AG), and PA_mid_TOPO (CAC CAA TTG AAG AAA GGT TTG). The amplified product was then subcloned into the pENTR/D-TOPO plasmid (Gateway®, Life Technologies™, Carlsbad, CA, USA) using topoisomerase I cloning. The resulting construct was then converted to pDEST10-PA using the clonase (Fig. 3A). The plasmid was transfected into Sf9 insect cells using the helper virus. The resultant baculovirus was again transfected into Sf9 cells [41]. The expressed full-length PA protein was purified using Ni^2+^-agarose and a HiTrap Q FF column (GE Healthcare) with the AKTA prime plus system (Fig. 3B).

### PA Endonuclease Activity and Fullerene Derivatives Nuclease Assays

Influenza A RNA polymerase PA endonuclease activity assays were performed as described by Dias et al. with some modifications [12]–[16]. Briefly, the pH was lowered from 8.0 to 7.3, and 1 µg M13 mp18 single-stranded circular phage DNA was used as the substrate. A total of 0.25 µg recombinant PA endonuclease domain or full-length PA protein were added to 100 µL assay buffer for each reaction (the final concentration of the protein was approximately 0.1 µM). For the fullerene derivative nuclease assay, no PA protein was added at this step. The fullerene derivatives (summarized in Fig. 1) were then added to the reaction, and the products were analyzed by agarose electrophoresis and stained with ethidium bromide.

### In Silico Docking Simulation Analysis of Fullerene C_60_ and the PA Endonuclease Domain of Influenza RNA Polymerase

All molecular modeling studies were performed using MOE software (Chemical Computing Group) [26], [42], [43]. Information regarding the tertiary structure of the influenza PA endonuclease domain (PDB ID: 3HW6) was obtained from a protein data bank [29]. This enzyme was prepared for docking studies in which (1) the ligand molecule was removed from the active site of the enzyme; (2) hydrogen atoms were added to the structure using standard geometry; (3) the structure was minimized using an MMFF94s force-field; (4) MOE Alpha Site Finder was used to search for active sites within the enzyme structure and dummy atoms were created from the obtained alpha spheres; and (5) the obtained model was then used in the Dock program (Ryoka Systems Inc., Tokyo, Japan). The conformation of the fullerene was generated by systematic, stochastic searches and Low Mode MD (molecular dynamics).

### MTT Cell Proliferation Assay

The cytotoxicity of the fullerene derivatives in MDCK cells was determined with an MTT cell proliferation assay kit according to the manufacturer’s instructions (Cayman, Arbor, MI, USA). Briefly, MDCK cells were cultured in Dulbecco’s modified Eagle medium (DMEM; Gibco/Invitrogen, Carlsbad, CA, USA) supplemented with 10% fetal bovine serum, 1% penicillin-streptomycin, and 4 mM L-glutamine at 37°C under 5% CO_2_. A confluent monolayer of MDCK cells was prepared in each well of a 96-well plate. Various concentrations (12.5–100 µM) of the fullerene derivatives and ME in DMSO (100 µM chemicals: 1%, 50 µM: 0.5%, 25 µM: 0.25%, 12.5 µM: 0.125%.), which were used as the anti-influenza activity [16], were mixed in an infection medium (DMEM supplemented with 1% bovine serum albumin, 1% penicillin-streptomycin, and 4 mM L-glutamine). The mixture was added to the cells, and the treated cells were incubated for 24 h at 37°C under 5% CO_2_. After incubation, the cells were treated with the MTT reagent and incubated for 4 h at 37°C under 5% CO_2._ The wells were treated with the crystal dissolving solution to lyse the formazan produced in the cells, and the absorbance of each well was measured at 570 nm using a microplate reader.

### Cytotoxicity Assay by Naphthol Blue Black

A confluent monolayer of MDCK cells was prepared in each well of a 96-well plate. Various concentrations (0.8–100 µM) of the fullerene derivatives in DMSO were mixed with an infection medium (DMEM supplemented with 1% bovine serum albumin, 1% penicillin-streptomycin, and 4 mM L-glutamine) and incubated for 30 min at 37°C under 5% CO_2_ [43]. The mixture was added to the cells, and the treated cells were incubated for 24 h at 37°C under 5% CO_2_. After incubation, the cells were fixed using a 10% formaldehyde solution. Viable cells were stained with a naphthol blue black solution (0.1% naphthol blue black, 0.1% sodium acetate, and 9% acetic acid) [43].

### Immunostaining of Influenza A Virus-infected Cells

MDCK cells were prepared in each well of a 96-well plate. Various concentrations (25–100 µM) of the fullerene derivatives and ME were mixed at a multiplicity of infection (MOI) of 1 influenza A virus (A/PR/8/34 (H1N1) or A/Aichi/2/68 (H3N2)) in the infection medium and incubated for 30 min at 37°C under 5% CO_2_. The mixture was added to the cells, and the treated cells were incubated for 24 h at 37°C under 5% CO_2_. After incubation, the cells were fixed with 4% paraformaldehyde in phosphate-buffered saline (-) for 30 min at 4°C and then permeabilized with 0.3% Triton X-100 for 20 min at room temperature. A mouse anti-influenza A NP antibody (FluA-NP 4F1; SouthernBiotech, Birmingham, AL, USA) and horseradish peroxidase-conjugated goat anti-mouse IgG antibody (SouthernBiotech) were used as primary and secondary antibodies, respectively [44]. To visualize the infected cells, TrueBlue peroxidase substrate (KPL, Gaithersburg, MD, USA) was added, and color development was terminated after 15 min of incubation by washing with H_2_O. The wells were photographed under a microscope, and the stained cells were counted.

### Western Blotting

MDCK cells were prepared in each well of a 24-well plate. We mixed 100 µM of the fullerene derivatives and ME at an MOI of 1 influenza A virus (A/PR/8/34 (H1N1)) in the infection medium and incubated the solution for 30 min at 37°C under 5% CO_2_. The mixture was added to the cells, and the treated cells were incubated for 4, 8, 12, and 24 h at 37°C under 5% CO_2_. After incubation, the cells were lysed with a sodium dodecyl sulfate buffer (125 mM Tris-HCl, pH 6.8, 5% sodium dodecyl sulfate, 25% glycerol, 0.1% bromophenol blue, and 10% β-mercaptoethanol) and boiled for 5 min. The cell lysates were then loaded onto a 10% polyacrylamide gel. The proteins were transferred to a polyvinylidene fluoride microporous membrane (Millipore, Billerica, MA, USA). For primary antibodies, mouse anti-influenza A NP antibody (FluA-NP 4F1; SouthernBiotech) and goat anti-influenza A NS1 antibody (vC-20; Santa Cruz Biotechnology, Santa Cruz, CA, USA) were used to detect NP and NS1, respectively. A rabbit anti-β–actin antibody (13E5; Cell Signaling, Danvers, MA, USA) was used as an internal control. Horseradish peroxidase-conjugated goat anti-mouse IgG antibody (SouthernBiotech), donkey anti-goat IgG antibody (sc-2020; Santa Cruz Biotechnology) and goat anti-rabbit IgG antibody (KPL) were used as secondary Abs. The blots were developed by using Western Lightning ECL Pro (PerkinElmer, Waltham, MA, USA).

### Statistical Analysis

All results were expressed as the mean ± standard error of the mean. Differences were analyzed for statistical significance by one-way analysis of variance (ANOVA) for comparison among the DMSO, ME and fullerene derivative-treated groups. The results were considered significantly different at *p<0.05 and **p<0.01 when comparing the number of stained cells in the ME or fullerene derivatives-treated groups to that of the DMSO-treated group.
