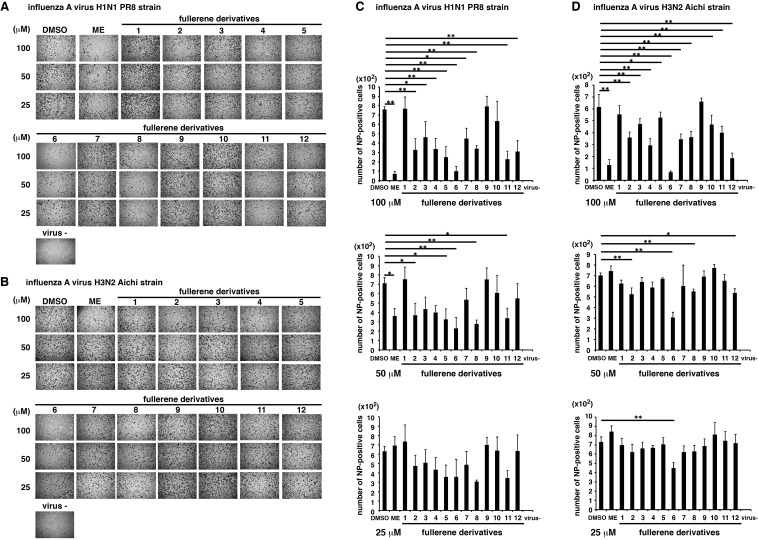# Correction: Anti-Influenza Activity of C_60_ Fullerene Derivatives

**DOI:** 10.1371/annotation/3e6e3fb0-e52f-4a6d-8ea2-34de4147b64f

**Published:** 2013-11-12

**Authors:** Masaki Shoji, Etsuhisa Takahashi, Dai Hatakeyama, Yuma Iwai, Yuka Morita, Riku Shirayama, Noriko Echigo, Hiroshi Kido, Shigeo Nakamura, Tadahiko Mashino, Takeshi Okutani, Takashi Kuzuhara

The µ symbol does not appear correctly in Figure 6 and 7. Please see the corrected figures here:

Figure 6: 

**Figure:**
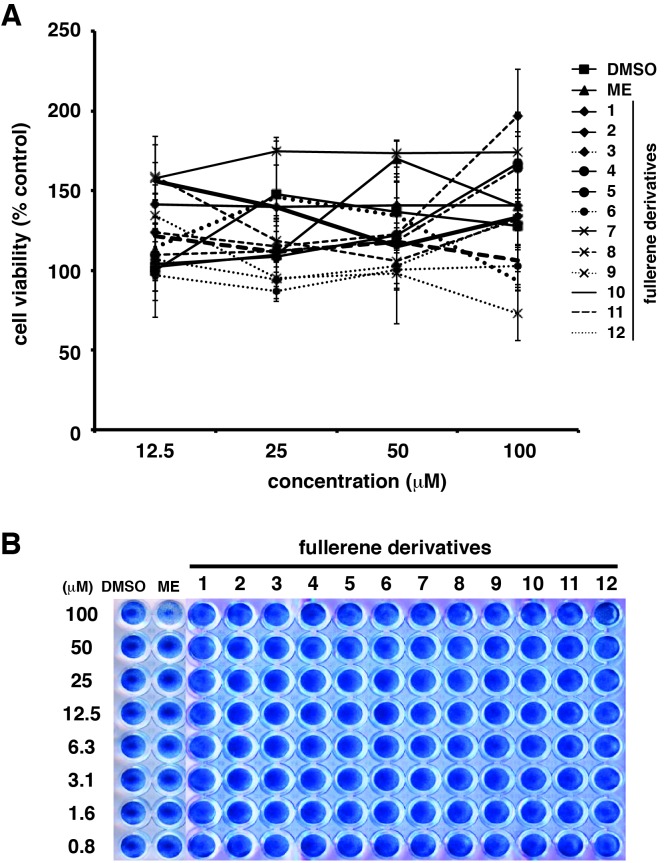


Figure 7: 

**Figure:**